# Rectal and axillary admission temperature in preterm infants less than 32 weeks' gestation, a prospective study

**DOI:** 10.3389/fped.2024.1431340

**Published:** 2024-07-05

**Authors:** Shaimaa Halabi, Rana Almuqati, Amenah Al Essa, Manal Althubaiti, Musab Alshareef, Radha Mahlangu, Abdulaziz Homedi, Faisal Alsehli, Saif Alsaif, Kamal Ali

**Affiliations:** ^1^ Neonatal Intensive Care Department, King Abdulaziz Medical City-Riyadh, Ministry of National Guard Health Affairs, Riyadh, Saudi Arabia; ^2^ King Abdullah International Medical Research Center, Riyadh, Saudi Arabia; ^3^ King Saud Bin Abdulaziz University for Health Sciences, Riyadh, Saudi Arabia

**Keywords:** preterm, temperature, axillary, rectal, correlation

## Abstract

**Objectives:**

The purpose of this research was to evaluate the differences between rectal and axillary temperature measurements in preterm infants who were born less than 32 weeks’ gestation using digital thermometers upon their admission to the Neonatal Intensive Care Unit (NICU).

**Methods:**

Prospective, observational, single centre study. Rectal and axillary temperatures measurements were performed using a digital thermometer. The study examined various maternal and neonatal factors to describe the study group, including the use of prenatal corticosteroids, the occurrence of maternal diabetes and hypertension, a history of maternal prolonged rupture of membranes (PROM), maternal chorioamnionitis, the mode of delivery, along with the neonate's gender, birth weight, and gestational age. The Pearson correlation coefficient (R) was calculated to ascertain the linear relationship between the temperatures taken at the rectal and axillary sites. The concordance between the two sets of temperature data was analyzed using the Bland-Altman method.

**Results:**

Eighty infants with a mean gestational age of 28.4 weeks (SD = 2.9) and a mean birth weight of 1,229 g (SD = 456) were included in the study. The mean axillary temperature was 36.4 °C (SD = 0.7), which was lower than the mean rectal temperature of 36.6 °C (SD = 0.6) (*p* = 0.012). Rectal temperatures surpassed axillary measurements in 59% of instances, while the reverse was observed in 21% of cases. Rectal and axillary temperatures had a strong correlation (Pearson correlation coefficient of 0.915, *p* < 0.001). Bland-Altman plot showed a small mean difference of 0.1C between the two temperatures measurements but the limits of agreement were wide (+0.7 to −0.6 °C). For hypothermic infants, the mean difference between rectal and axillary temperatures was 0.27 °C, with a wide limit of agreement ranging from −0.5 °C to +1 °C. Conversely, for normothermic infants, the mean difference was smaller at 0.1 °C, with a narrower limit of agreement from −0.4 °C to +0.6 °C.

**Conclusions:**

While there is a good correlation between axillary and rectal temperatures, the wider limits of agreement indicate variability, particularly in hypothermic infants. For a more accurate assessment of core body temperature in hypothermic infants, clinicians should consider using rectal measurements to ensure effective thermal regulation and better clinical outcomes.

## Introduction

Based on the World Health Organization (WHO) practical guide on thermal protection of the newborn published in 1997, the temperature ranges for hypothermia and normothermia in neonates are as follows: Mild hypothermia is defined as a temperature between 36.0 °C and 36.4 °C, moderate hypothermia between 32.0 °C and 35.9 °C, and severe hypothermia below 32.0 °C. Normothermia is defined as a temperature between 36.5 °C and 37.5 °C. Preterm infants are particularly prone to rapid heat loss following birth. Despite efforts to mitigate heat loss during stabilization in the delivery room (DR), a significant number of preterm newborns still present with abnormal temperatures upon admission to the neonatal intensive care unit (NICU) (1, 2).

Previous research has indicated that that hypothermia at admission is linked to a higher likelihood of mortality and various complications in newborns (3–8).

There is no definitive standard for measuring newborn's body temperature, but ideally, clinicians should measure rectal temperature (RT) to accurately reflect the body's core temperature, particularly the hypothalamic temperature (9). Previous studies using glass and mercury thermometers have highlighted potential complications associated with measuring rectal temperature in infants (10, 11). To prevent these complications, axillary temperature measurements are commonly used as a surrogate measure of the core body temperature. Measuring the temperature from the axilla is easier to access and is less invasive compared to the rectum.

Inconsistent results have been noted in studies assessing the agreement between rectal and axillary temperatures taken with digital thermometers in newborns, infants and children. A meta-analysis that reviewed 20 studies involving 3,201 term infants and children found notable discrepancies in temperature measurements (12). The overall mean difference between rectal and axillary temperatures was 0.85 °C, with a variation range from −0.19 °C to 1.90 °C, showing a wide degree of variability (12). For newborns specifically, this variability was smaller, with a mean difference of 0.17 °C, which varied from −0.15 °C to 0.50 °C (12).

However, much of the existing research on rectal and axillary temperature correlation is centered on comparing rectal and axillary temperature measurements in stable, normothermic infants who are past the admission period—the first hour after birth—at various times during their stay in NICU. Additionally, the majority of previous studies only compared the AT and RT measurements in term or late preterm infants. Therefore, we focused on extremely and very preterm infants <32 weeks of gestational age to determine whether AT and RT measurements are also comparable in this group of patients. Furthermore, a notable gap exists in understanding this correlation specifically among newborn very preterm infants upon admission post-stabilization in the delivery room. This population often presents unique challenges due to deviations from normothermia and a lack of clinical stability. Consequently, there is a pressing need to investigate the correlation between rectal and axillary temperatures in this cohort.

The purpose of this research was to evaluate the differences between rectal and axillary temperature measurements in preterm infants less than 32 weeks' gestation upon admission to the NICU.

## Methods

Between the 27th of September 2023 and the 31st of March 2024, a prospective observational study was carried out in the NICU at The King Abdulaziz Medical City (KAMC), Riyadh, Kingdom of Saudi Arabia. We included premature infants who were less than 32 weeks gestational age at birth. Outborn infants and those with birth defects that made it impossible to measure temperature via the axilla or the rectum were excluded.

King Abdullah International Medical Research Centre (KAIMRC) ethics committee approved the project with IRB number: NRC23R/336/04. Informed consent was obtained retrospectively in cases of emergent deliveries where antenatal consent could not be obtained. This deferred consent process allowed us to enroll patients and collect data while ensuring that parents were approached for consent at the earliest appropriate opportunity.

Temperature in the operating theatre (OT) and the DR was kept at between 23 and 23.5 °C. Directly following birth, infants were promptly positioned in a plastic covering and a hat was applied to cover the head. The plastic bag used in our study is NeoHelp™ by Vygon. NeoHelp™ is made of transparent polyethylene, which allows for visual monitoring of the infant while maintaining a warm environment. The portable incubator (Babyleo TN500; Drager) utilized for transferring the infants between the DR/OT and the NICU was preset to a stable temperature of 36 °C. To ensure optimum warmth, the NICU's incubators were conditioned with moisture and heat before each delivery with the humidity level set at 80%. The NICU is directly opposite the DR and OT.

Upon the infants' entry into the NICU, after initial medical care and taking body size measurements, their admission temperatures were recorded within the first hour after birth.

Temperature measurements of both the rectum and axilla were taken using the “Safety 1st 3-in-1 Nursery Thermometer,” which is approved for rectal and axillary use in children and infants. The Thermometer is manufactured by Dorel Juvenile. The measurement temperature range for this thermometer is 32.2–43.2 °C. It has a measurement accuracy of ±0.1 °C for temperatures between 35 and 42 °C and ±0.2 °C for temperatures below 35 °C and above 42 °C. The operational environment for the thermometer is between 10 and 40 °C, and the storage environment is between −25 °C and 60 °C.The manufacturer states that this thermometer is calibrated at the time of its production, and adhering to the provided instructions ensures that the measurement accuracy remains intact. It is a digital thermometer that beeps when the reading is complete. It has a flexible tip which is comfortable for infants during a rectal reading and features an over-insertion gauge for safety during rectal use. The 3-in-1 Nursery Thermometer includes both a protective storage case and a long battery life. It provides accurate reading in 30 s (°F/C) and has the feature to recall last reading.

Eight medical staff were involved in the study and undertook training on temperature measurement using the digital thermometer on the two sites. This helped to ensure the validity of the rectal and axillary temperature measurements in infants included in the study. In every case, rectal temperature was recorded first. Members of the research team were instructed to turn on the thermometer and wait for a signal before accessing the incubator through the portholes. They were then to gently insert the thermometer's tip up to 1 cm deep into the rectum. The device continuously displayed the temperature until a second signal (eight beeps) indicated that the peak temperature was achieved, usually within about 30 s. For axillary temperature, the thermometer was placed under the arm, ensuring the tip contacted the skin, following the previously outlined procedure. The same thermometer was used for all measurements and was cleaned with an alcohol wipe between each use to maintain hygiene and accuracy.

The study examined various maternal and neonatal factors to describe the study group, including maternal prenatal corticosteroid administration, the occurrence of both pre-pregnancy diabetes mellitus (DM) and gestational diabetes mellitus (GDM), hypertension (both pre pregnancy and pregnancy induced), a history of prolonged rupture of membranes (PROM) in the mother, history of chorioamnionitis, the mode of delivery, along with the neonate's gender, birth weight, and gestational age. In our study, chorioamnionitis was defined clinically based on the presence of maternal fever (≥38.0 °C) and one or more of the following criteria: uterine tenderness, maternal or fetal tachycardia, foul-smelling amniotic fluid, or purulent vaginal discharge, as per the guidelines recommended by the American College of Obstetricians and Gynecologists (13). In our study, antenatal steroid administration was reported for any administration of steroids, including both partial and full courses. At our center, dexamethasone is used as the corticosteroid of choice.

## Statistical analysis

The statistical evaluation of the data was conducted using the SPSS version 26.0 software. For this study, a power analysis factoring in the projected differences in rectal and axillary admission temperatures and the standard deviations from existing literature (14) indicated that a sample size of 80 infants would be sufficient. With a targeted power level of 80% and a significance level set at 0.05, this number of participants was anticipated to allow for the detection of statistically significant differences in temperature measurements between the two methods in infants born at less than 32 weeks' gestation.

Normality test was done using the Kolmogorov-Smirnov test. Continuous variables that displayed a normal distribution, were presented as the average (mean) with the standard deviation (SD). On the other hand, variables not adhering to a normal distribution were expressed using the median and the interquartile range (IQR). The comparison of rectal and axillary temperature readings utilized the student's paired t-test, with a *p*-value threshold of less than 0.05 set for determining statistical significance. Variables that were categorical in nature were represented as frequencies and percentages. The Pearson correlation coefficient (R) was calculated to ascertain the linear relationship between the temperature taken at the rectal and axillary sites, and this correlation was illustrated through a scatterplot. The concordance between the two sets of temperature data was analyzed using the Bland-Altman method, which involved plotting the difference between the axillary and rectal readings against their average value. The consistency range was defined by the mean difference plus or minus two standard deviations, establishing the bounds of agreement (15).

## Results

In this study, we included eighty infants with a mean gestational age of 28.4 weeks (SD = 2.9) and a mean birth weight of 1,229 grams (SD = 456). The majority of the infants (59%) were male, and (62.5%) were delivered via caesarean section. Antenatal steroid therapy was administered in 74% of the cases. Maternal health conditions were documented: 25% of the mothers had diabetes, 12.5% had hypertension, 38.8% experienced prolonged rupture of membranes (PROM) lasting more than 18 h, and chorioamnionitis was present in 8.8% of the cases (Table 1).

**Table 1:** Maternal and infants’ characteristics.

Gestational age (weeks) (Mean ± SD)	28.4 (2.9)
Birthweight (grams) (Mean ± SD)	1,229 (456)
Gender (male)	47/80 (59%)
Antenatal steroids	59/80 (74%)
Delivery by Caesarean section	50/80 (62.5%)
Maternal hypertension	10/80 (12.5%)
Maternal diabetes	20/80 (25%)
Maternal Prolonged Rupture of Membranes (PROM) > 18 h	31/80 (38.8%)
Maternal chorioamnionitis	7/80 (8.8%)

Table 2 presents the temperature data for both mothers and infants participating in the study. The ambient temperature in the DR/OT averaged 23.2 °C (SD = 0.2), while maternal axillary temperature at the time of birth was 36.8 °C (SD = 0.3). In line with hypothermia prevention protocols, all neonates were promptly covered in plastic wrap and fitted with head covers post-delivery. The infants were admitted to the NICU within 17 min (SD = 9) of birth. Upon NICU admission, the mean axillary temperature registered at 36.4 °C (SD = 0.7), which was significantly lower than the mean rectal temperature which was 36.6 °C (SD = 0.6) (*p* = 0.012). The range of axillary temperature was 5.2 °C (33.2–38.4 °C), whereas the range for rectal temperature was 4.5 °C (33.9–38.4 °C). Rectal temperature surpassed axillary measurements in 59% of instances, while the reverse—axillary readings higher than rectal—was observed in 21% of cases. In 20% of the occurrences, both temperature readings were analogous. No rectal injuries were reported among the patients in our cohort.

**Table 2:** Maternal and infant's temperatures characteristics.

Maternal axillary temperature °C (Mean ± SD)	36.8 (0.3) °C
DR/OT temperature °C (Mean ± SD)	23.2 (0.2) °C
Use of plastic bag (NeoHelp™)	80/80 (100%)
Use of head cover	80/80 (100%)
Time from birth to admission (minutes) (Mean ± SD)	17 (9)
Time from birth to admission (minutes) for (DR) deliveries (Mean ± SD)	17 (9)
Time from birth to admission (minutes) (OT) (Mean ± SD)	18 (8)
Infant's axillary temperature °C (Mean ± SD)	36.4 (0.7)
Infants’ rectal temperature °C (Mean ± SD)	36.6 (0.6)
Rectal temperature > axillary	47/80 (59%)
Rectal temperature < axillary	17/80 (21%)
Rectal temperature = axillary	16/80 (20%)

DR = Delivery Room. OT = Operating Theatre.

**Figure 1:**
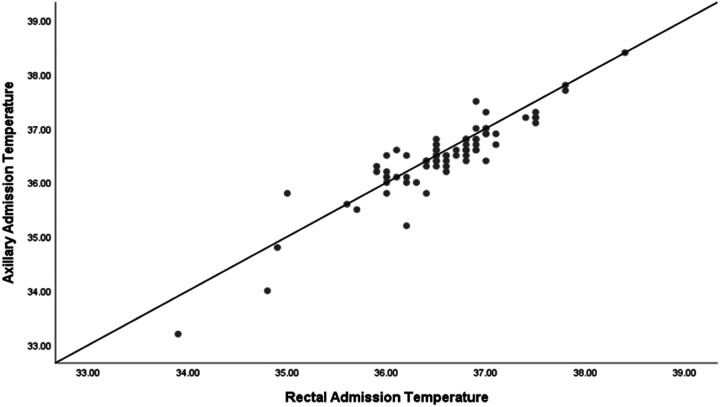
Scatter plot of rectal and axillary admission temperature.

This scatter plot (Figure 1) depicts the correlation between rectal and axillary admission temperature in our cohort. The data points represent individual temperature pairs measured upon admission to the NICU, with rectal temperature along the x-axis and axillary temperature along the y-axis. Our analysis of the correlation between rectal and axillary admission temperatures in our cohort yielded a Pearson correlation coefficient of 0.915, which is highly significant (*p* < 0.001) (Table 3). This strong positive correlation is illustrated in the scatter plot, where the close clustering of data points around the line of best fit indicates a consistent linear relationship between the rectal and axillary temperature (Figure 1).

**Table 3:** Correlation of rectal and axillary admission temperature.

		Axillary temperature	Rectal temperature
Axillary temperature	Pearson correlation	1	0.915**
	Sig. (2-tailed)		<0.001
	*N*	80	80
Rectal temperature	Pearson correlation	0.915**	1
	Sig. (2-tailed)	<0.001	
	*N*	80	80

** Correlation is significant at the 0.01 level (2-tailed).

**Figure 2:**
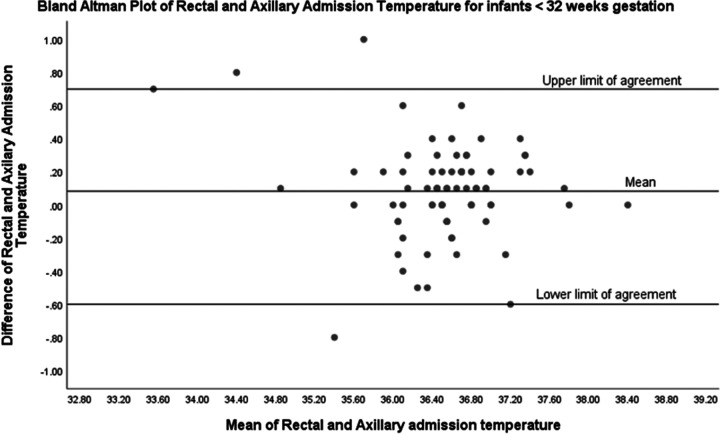
Bland-Altman plot of rectal and axillary temperature (*n* = 80).

In evaluating the clinical agreement between rectal and axillary temperature measurements in preterm infants, a Bland-Altman plot was employed. This plot revealed a mean difference, or bias, of 0.1 °C, suggesting a slight tendency for axillary temperatures to measure marginally lower than rectal temperatures. Clinically, this minimal bias may be considered negligible; however, the scope of agreement requires careful consideration. The limits of agreement, calculated as the mean difference plus and minus 1.96 times the standard deviation of the differences, range from +0.7 to −0.6 °C (Figure 2). This range is relatively wide, particularly in the context of the narrow temperature range that is critical in neonatal care.

**Figure 3:**
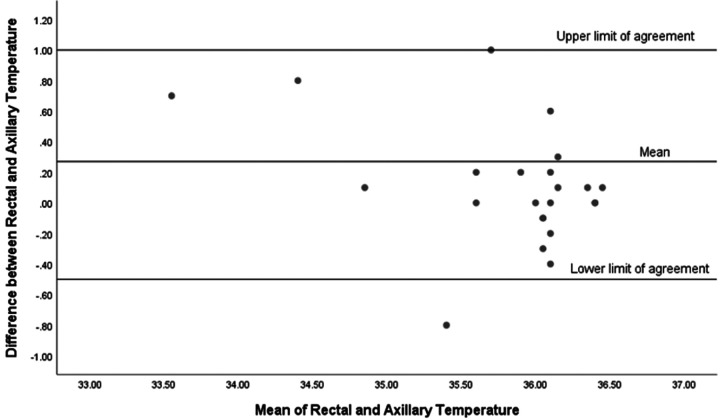
Bland-Altman plot for infants with admission temperature <36.5  °C (*n* = 23).

**Figure 4:**
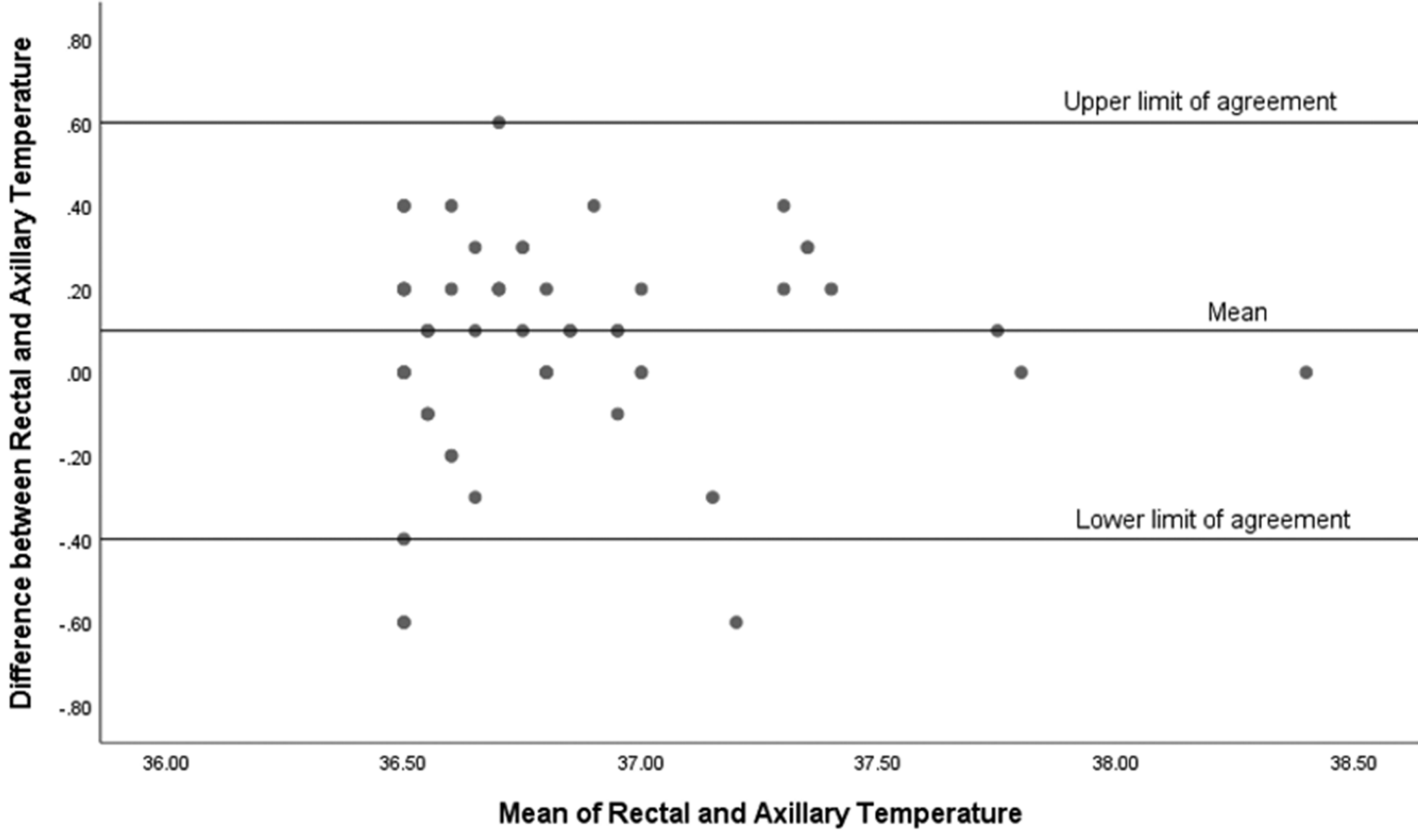
Bland-Altman plot for infants with admission temperature >36.5 °C (*n* = 57).

We have analyzed the rates of hypothermia on admission to the NICU (<36.5 °C) in our cohort and found that 23 out of 80 infants (29%) were hypothermic upon admission. Additionally, we examined the limits of agreement between the two temperature measurements for both hypothermic and normothermic infants and found notable differences. For infants who were hypothermic on admission to the NICU (*n* = 23), the mean difference between the two temperature measurements was 0.27 °C, with limits of agreement ranging from −0.5 °C to +1 °C (Figure 3). This indicates a wider range of discrepancy between the temperature measurements for hypothermic infants. In contrast, for infants with normal admission temperatures (*n* = 57), the mean difference was smaller at 0.1 °C, with a narrower limit of agreement ranging from −0.4 °C to +0.6 °C (Figure 4).

## Discussion

In this prospective study, we observed a strong correlation between rectal and axillary temperature measurements in preterm infants who were born <32 weeks' gestation. Additionally, we found a high incidence of hypothermia on admission in these infants, with 29% being hypothermic upon arrival at the NICU. Despite the strong correlation between rectal and axillary temperatures, rectal temperatures were consistently higher than axillary temperatures, raising important considerations for clinical practice. Nevertheless, the limits of agreement between these two measurement methods were wide, particularly in infants who were hypothermic upon admission. This discrepancy highlights the potential challenges in relying solely on axillary temperatures for clinical decisions in the NICU.

In our study, the infants were predominantly delivered via cesarean section, had a mean gestational age of 28.4 weeks and received immediate interventions to prevent hypothermia in the delivery room as per the Newborn Resuscitation Program (NRP) (16).

We have shown that rectal temperatures in our cohort were higher than the axillary temperatures. Despite the strong correlation observed in the scatter plot analysis, the Bland-Altman analysis provided additional insight into the clinical applicability of using axillary temperature as a surrogate for rectal temperature. Our Bland-Altman plot for all infants in our cohort revealed that although the mean difference between rectal and axillary temperature measurements was small (0.1 °C), indicating good initial agreement between the two methods, the limits of agreement were wide (−0.6 °C to +0.7 °C). This discrepancy was even more pronounced in hypothermic infants. For hypothermic infants, the mean difference between rectal and axillary temperatures was 0.27 °C, with a wide limit of agreement ranging from −0.5 °C to +1 °C. This significant discrepancy suggests that axillary temperatures may not reliably reflect core body temperatures in hypothermic infants, potentially leading to suboptimal clinical decisions. Conversely, for normothermic infants, the mean difference was smaller at 0.1 °C, with a narrower limit of agreement from −0.4 °C to +0.6 °C, indicating a more consistent correlation between the two measurement methods. Therefore, while axillary temperature measurements may be informative for population-level assessments due to their correlation with rectal temperatures, our results advise caution when applying these findings to individual neonates. These findings emphasize the importance of selecting the appropriate method for temperature measurement in the NICU. While axillary temperature measurements are less invasive and generally correlate well with rectal temperatures in normothermic infants, their reliability decreases in hypothermic infants. Therefore, clinicians should consider using rectal temperature measurements for a more accurate assessment of core body temperature in hypothermic infants to ensure effective thermal regulation and better clinical outcomes.

The average discrepancy noted between rectal and axillary temperature readings of 0.1 °C. stands in contrast to the larger differences between rectal and axillary temperatures documented in earlier studies, where mean differences were observed at 0.17 °C (12), 0.27 °C (14), 0.16 °C (17), and significantly, a 0.7 °C difference (18). A plausible reason for our study showing a narrower margin could be the uniformity of our sample group, which consisted solely of preterm infants less than 32 weeks' gestation, and the fact that temperature measurements were conducted concurrently at admission to NICU within a narrow time window of one hour. Other studies have incorporated a broader and more varied demographic, such as one investigation that looked at infants aged up to six months in both hospital and home environments (18), while another recorded temperatures at various intervals during the infants' stay in the NICU (17). Additionally, some studies have combined data from both term and preterm infants in their analysis (12, 14). In one particular study that performed a subgroup analysis on preterm infants, the data suggested that the alignment between rectal and axillary temperatures tends to be more consistent in preterm infants compared to those who are term (17).

More importantly, there is a scarcity of data concerning the correlation of rectal and axillary temperatures of extremely and very preterm infants at the time of their NICU admission. In a similar study to ours published recently (19), the authors found the mean difference between the rectal and axillary temperatures to be the same as ours (0.1 °C). However, the limits of agreement were even wider than ours (−1.4 °C-+1.5 °C). This suggests a tighter agreement in our measurements, yet it still underscores the potential clinical implications when utilizing axillary temperatures as proxies for rectal measurements in this patient population. We speculate that differences in the demographic characteristics of the study populations, such as age, weight, and clinical conditions, might have played a role in the different level of agreement between the two studies. Additionally, the types of thermometers used in the two studies were different, affecting the accuracy and consistency of the measurements. Finally, environmental conditions during temperature measurements, such as room temperature and humidity, could have influenced the results.

A notable advantage of our investigation is the provision of prospective data on a comparably uniform and sizeable cohort of extremely and very preterm infants, all assessed at the same temporal juncture. We recorded the rectal and axillary temperatures sequentially with the same thermometer for each baby, without any intervening medical procedures to modify the infant's body heat, making it improbable that body temperature fluctuations contributed to the observed differences in readings. Nevertheless, our study is not without its limitations. Despite utilizing a uniform thermometer for recording both rectal and axillary temperatures, the measurements were taken by various nurses and doctors, each with different levels of expertise. Nonetheless, the consistency of the thermometers and adherence to standard clinical procedures and manufacturer guidelines ensure a level of standardization. Still, numerous potential issues can affect the accuracy of temperature readings. The precision of axillary temperature can be influenced by factors such as the proper positioning of the axilla and local blood circulation. Similarly, the accuracy of rectal temperature readings might be impacted by how deeply the thermometer is inserted and whether there is stool in the rectum. Finally, this study is limited by its single-center design and relatively small sample size, which may restrict the generalizability of our findings. Future studies should aim to include multiple centers to enhance the robustness and applicability of the results.

Occasionally, there's hesitancy in performing rectal temperature checks on newborns due to the perception of it being an invasive procedure, historically linked to rare instances of rectal perforation. However, the incidence of injury from such temperature assessments is noted to be exceedingly low (18), with most reported cases of injury pertaining to the now-out of use mercury-in-glass thermometers. Contemporary practice has shifted to using plastic, digital thermometers that are designed with slender, smooth probes that produce results in just 30 s, diminishing the likelihood of breakage and injury.

Our current practice for measuring temperature in preterm infants at admission involves using axillary temperature measurements due to their non-invasive nature and ease of use. Although our study provides valuable insights, these findings are not yet sufficient to change our practice. Further validation through larger multicenter trials is necessary to establish robust evidence that could potentially influence clinical guidelines.

## Conclusion

Given the critical importance of accurate thermal regulation in preterm infants, our study highlights the need for cautious interpretation of axillary temperature measurements upon admission to the NICU. While we found a strong correlation between rectal and axillary temperatures, the limits of agreement were wide, particularly in hypothermic infants. Therefore, clinicians should be aware of these discrepancies and consider using rectal temperature measurements, especially in infants with hypothermia, for more accurate assessment of core body temperature. This approach will help ensure more reliable data for critical care decisions, ultimately improving outcomes for premature infants.

## Data Availability

The raw data supporting the conclusions of this article will be made available by the authors, without undue reservation.
